# Effect of Experimental Parameters on Morphological, Mechanical and Hydrophobic Properties of Electrospun Polystyrene Fibers

**DOI:** 10.3390/ma8052718

**Published:** 2015-05-20

**Authors:** Siqi Huan, Guoxiang Liu, Guangping Han, Wanli Cheng, Zongying Fu, Qinglin Wu, Qingwen Wang

**Affiliations:** 1 College of Material Science and Engineering, Northeast Forestry University, Harbin 150040, China; E-Mails: huangsiqi888@hotmail.com (S.H.); gxliunefu@hotmail.com (G.L.); wonwinfu@hotmail.com (Z.F.); qwwang@nefu.edu.cn (Q.W.); 2 School of Renewable Natural Resources, Louisiana State University Agricultural Center, Baton Rouge, LA 70803, USA; E-Mail: qwu@agcenter.lsu.edu

**Keywords:** electrospinning, polystyrene nanofiber, morphology, tensile strength, hydrophobicity

## Abstract

Polystyrene (PS) dissolved in a mixture of *N*, *N*-dimethylformamide (DMF) and/or tetrahydrofuran (THF) was electrospun to prepare fibers with sub-micron diameters. The effects of electrospinning parameters, including solvent combinations, polymer concentrations, applied voltage on fiber morphology, as well as tensile and hydrophobic properties of the fiber mats were investigated. Scanning electron microscope (SEM) images of electrospun fibers (23% w/v PS solution with applied voltage of 15 kV) showed that a new type of fiber with double-strand morphology was formed when the mass ratio of DMF and THF was 50/50 and 25/75. The tensile strength of the PS fiber film was 1.5 MPa, indicating strong reinforcement from double-strand fibers. Bead-free fibers were obtained by electrospinning 40% (w/v) PS/DMF solution at an applied voltage of 15 kV. Notably, when the ratio of DMF and THF was 100/0, the maximum contact angle (CA) value of the electrospun PS films produced at 15 kV was 148°.

## 1. Introduction

One-dimensional (1D) nanostructures in the form of fibers, wires, rods, belts, tubes, and rings have attracted interest due to their novel properties and intriguing applications in many areas. Significant advances in the fabrication of 1D nanostructures with well-controlled morphology and chemical composition have been made using a number of manufacturing procedures [1], including drawing, template synthesis, phase separation, self-assembly, and electrospinning, which can be adopted to prepare nanofibers based on polymers, metals, ceramics, and glass. Moreover, the assembly of nanofibers into two-dimensional (2D) and three-dimensional (3D) nanostructures has also been applied to various practical applications [2]. Among these methods, electrospinning, as one of the most versatile techniques for fabricating ultrafine non-woven polymer fibers, has been paid considerable attention in recent decades due to its simplicity, high efficiency, and low cost [3,4,5]. The obtained mats composed of electrospun nanofibers have several advantageous characteristics such as small diameters, neat morphology, and an interconnected network structure [6]. These outstanding properties make the electrospun mats ideal candidates for different applications, e.g.*,* filtration [7], wound dressing [8], and protective clothing [9]. In particular, polymer mats with superhydrophobicity manufactured by electrospinning have been widely investigated in the field of self-cleaning materials and high-performance coatings [10,11].

Polystyrene (PS) is a class of low cost, promising thermoplastic polymers. The inherent hydrophobicity of PS has also been extensively explored and utilized to create superhydrophobic materials with tailored performances [12]. Nevertheless, the applications of PS films produced with traditional methods are limited in many aspects due to their brittleness. Moreover, in order to improve the film-forming ability of PS, film-forming aids are commonly added during the film manufacturing process, which can lead to volatile organic compound (VOC) emission which pollutes the environment and is harmful to human health. Therefore, the desirability to improve the shortcomings of PS materials is becoming increasingly important and various approaches have been implemented through physical and chemical modifications [13,14,15,16]. However, such approaches are either limited to creating PS materials with specific components, or which require a complex fabricating process. In fact, no simple PS modified formulation is commercially available that can offer the potential to overcome all the drawbacks of PS materials. Therefore, the development of simple, environmental friendly methods to prepare high-performance PS materials is critical.

Electrospinning potentially has great utility as a novel manufacturing technique to enhance the properties of PS materials. Numerous research works have been published to study the fabrication and properties of electrospun PS mats [17,18,19]. Zhan *et al.* [20] manufactured electrospun PS mats with good tensile properties and different morphologies. Uyar *et al.* [21] considered the effect of solvent conductivity and produced reproducible uniform electrospun nanofibers for optimal electrospinning conditions. Their results showed that solutions with a higher conductivity yielded bead-free fibers from lower polymer concentrations [21]. In addition, another potential advantage for PS materials manufactured by electrospinning is to realize multi-functionalities by tuning the surface structure of nanofibrous mats, which opens a new avenue to create superhydrophobic PS materials [22].

During the process of electrospinning, the liquid drop elongates with the increasing electric field and distorts into a conical shape when the repulsive force is equal to the surface tension of the liquid. The fluid extension first occurs uniformly as straight flow lines, and then undergoes a vigorous whipping motion caused by electrohydrodynamic instabilities. As the solvent in the jet solution evaporates, the polymer fibers are collected onto a grounded substrate to form a non-woven mat or network. Based on previous reports [23], beads, necklaces, as well as ribbon-like and branched jet can be formed during the process of electrospinning PS with different experimental parameters. These parameters include spinning solution properties (e.g., viscosity, conductivity, and surface tension), governing variables (e.g., hydrostatic pressure in the capillary tube, electric potential at the capillary tip, and the distance between the tip and the collecting screen), together with ambient parameters (e.g., temperature, humidity, and air velocity in the electrospinning chamber) [24]. However, the conditions for the electrospinning process of PS solutions may be affected mutually, leading to uncontrollable PS fiber morphologies. Therefore, it is necessary to explore the effect of electrospinning parameters on the morphologies of PS fibers, offering potential to controllably produce electrospun PS fibers. Furthermore, it is well known that the properties of electrospun fibers are largely dependent upon the morphologies and surface structures of fibers [25]. Therefore, a fundamental understanding of how to regulate the morphology of electrospun PS fibers is also a prerequisite for manufacturing electrospun PS nanofibrous mats with high performances and novel functionalities. Additionally, determining the link between electrospinning parameters and fiber morphologies will further allow for sophisticated design and precise control of polymeric mats to meet specific application needs.

**Figure 1:**
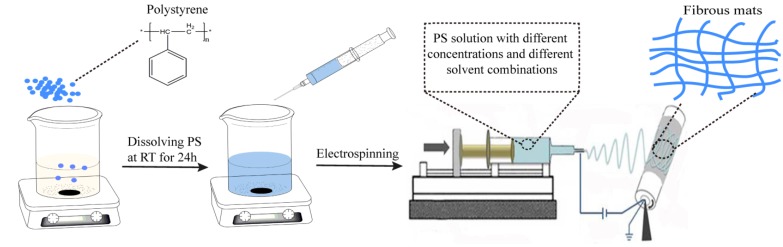
Schematic illustration of electrospinning process of polystyrene (PS) solutions and manufactured PS nanofibrous mats.

In the present work, PS nanofibrous mats were manufactured by electrospinning PS solutions (Figure 1). *N,N*-dimethylformamide (DMF) and tetrahydrofuran (THF) were used as solvents for preparing PS solutions with different solvent combinations and PS concentrations. The physiochemical properties (e.g., viscosity, surface tension, and conductivity) of these solutions were characterized. The effects of solvents, solution concentrations, and applied voltage on the morphological appearance of the obtained PS beads and 1D fibers were investigated using scanning electron microscope (SEM). The contact angle and tensile property were characterized to explore further application of the electrospun PS nanofibers as superhydrophobic nanofibrous mats. Most importantly, a new type of controllable parallel double-strand PS fibers with high hydrophobicity was successfully produced using a mixed solvent of DMF and THF at the ratio of 50/50 and 25/75 at a solution concentration of 23% (w/v). To our knowledge, the fabrication and control of special morphological PS fibers, especially for the parallel double-strain structure in this paper, have not been fully investigated.

## 2. Experimental

### 2.1. Materials

PS particles (melt flow rate = 4.0 g/10 min) with average molecular weight of 260,000 were purchased from Aladdin (Shanghai, China). The solvents used were a mixture of DMF (AR grade, Kermel Co., Tianjin, China) and THF (AR grade, Kermel Co., Tianjin, China). These materials were used as received without further purification.

### 2.2. Preparation of As-Spun Solutions

Different types of solvents for electrospinning were prepared by using a mixture of DMF and THF in the weight ratios of 100/0, 75/25, 50/50, 25/75 and 0/100 of 23% (w/v). Polymer solutions in DMF with 10, 23, 27, 32, 40% (w/v) concentrations were made simultaneously. PS particles were dissolved in the given solvents at room temperature (25 ± 2 °C) with 12 h stirring to obtain homogeneous solutions.

### 2.3. Characterization of Electrospinning Solutions

Viscosity, surface tension and conductivity of the PS solutions were characterized at room temperature by using a digital rotational viscometer (SNB-1, Heng Ping Co., Shanghai, China), surface tension meter (JK 98B, Shanghai, China), and conductivity meter (DDSJ-318, Lei Ci Co., Shanghai, China), respectively.

Viscoelastic properties of PS solutions were carried out using an AR2000ex (TA Instruments) controlled strain rheometer equipped with a 40 mm Teflon plate-and-plate geometry. The gap was fixed at 1 mm. Sample edges were covered with paraffin oil to prevent evaporation during measurements. The mechanical spectrum, storage modulus (G') and loss modulus (G"), were obtained at room temperature in the range of 0.1–100 Hz. Three replicates prepared at each composition were measured.

### 2.4. Electrospinning Set-up

The electrospinning apparatus (Yong Kang Le Ye Co., Beijing, China) is composed of a 5-mL syringe (Zhi Yu Co., Shanghai, China) connected to a syringe pump that was encased in a vented Plexiglas box [950 mm (L) × 820 mm (W) × 950 mm (H)] (Figure 1). The syringe pump was used to supply a steady flow of 0.0625 mL·min^−1^ of solution to the tip of the needle. A high-voltage power supply was used to apply a potential of 10, 15, 20 kV to the syringe needle. The needle used was size 22 and the corresponding inner diameter was 0.4 mm. A grounded cylinder collector (340 mm in length and 108 mm in diameter) was rotated at a speed of 80 rpm and placed 15 cm apart from the needle tip to test the distances at which the fibers were dry upon collection. A rectangular piece of aluminum foil (240 mm in length and 150 mm in width) was used to cover the cylinder to collect nonwovens of the electrospun nanofibers. The relative humidity and temperature were measured by a hygrothermograph placed inside the electrospinning chamber. They were kept at constant values of 13% and 25 °C, respectively.

### 2.5. Characterization of Electrospun PS Fibers

Scanning electron microscope (SEM, QUANTA-200, FEI, Hillsboro, OR, USA) was used to obtain microphotographs of the non-woven nanofibers formed after electrospinning. The nanofibrous mats were collected on the aluminum foil. The mats were cut into small pieces, and then coated with a layer of gold-palladium before being observed with SEM at an accelerating voltage of 12.5 kV. The diameter and distribution of the electrospun nanofibers were analyzed from the SEM images by using Nanometer software (Fudan University, China). At least ten beads were measured for the aspect ratio (major axis: bead length along the fibers; minor axis: bead length perpendicular to the fiber) and 100 fibers were measured to obtain the average fiber diameter.

The wettability of the PS surface was characterized on a contact angle meter (OCA20, Dataphysics, Bad Vilbel, Germany) at ambient conditions. Contact angle (CA) was measured using a sessile drop method. A droplet of 5 μL volume was used. The CA values of the right side and the left side of the water droplet were both measured and averaged. All the CA data were an average of five measurements at different locations on the surface.

Dynamic mechanical properties of the electrospun PS mats were measured in tensile mode using a dynamic mechanical analyzer (DMA, TA Instruments Q800). The measurements were performed at a constant frequency of 1 Hz and strain amplitude of 0.01%, for temperature range of room temperature to 150 °C, using a heating rate of 5 °C/min and a gap between jaws of 10 mm. Three samples were used to characterize each material.

Mechanical properties of the electrospun mats were determined from the stress-strain curves from tensile tests. The tensile test of the samples was carried out on a Model 3365universal testing machine (Instron Co., Norwood, MA, USA) with a tensile rate of 50 mm·min^−1^ according to ASTM D 882-09 at room temperature and 30% humidity. The size of each sample was of 15 mm length and 5 mm width. Three replicates of the mats prepared at each composition were measured.

## 3. Results and Discussion

### 3.1. Fiber Morphology

#### 3.1.1. Effect of Solvent Types

In the electrospinning process of a polymer solution, the solvent is one of the main contributors for solution properties, e.g., conductivity, surface tension, and viscosity [26]. Hence, the morphology of electrospun fibers can be altered by changing the solvent composition to control the surface tension and viscosity of solutions at constant solution concentration. The properties of the prepared solvents and solutions are summarized in Table 1 and Table 2, respectively.

**Table 1:** Basic properties of solvents ^a^.

Solvent	Viscosity (mPa·s)	Surface tension (mN·m^−1^)	Conductivity (F·m^−1^)	Boiling point (°C)
DMF	0.80	35.20	0.55	152.8
DMF/THF (75/25)	0.73	31.34	0.47	—
DMF/THF (50/50)	0.67	30.80	0.43	—
DMF/THF (25/75)	0.61	28.60	0.35	—
THF	0.53	26.40	—	66

^a^ Value reported for 25 °C. The ratio represented the weight ratio of dimethylformamide (DMF) to tetrahydrofuran (THF).

**Table 2:** Characteristic properties of tested solutions ^a^.

Solution	Concentration (w/v, %)	Viscosity (mPa·s)	Surface tension (mN·m^−1^)	Conductivity (F·m^−1^)
PS/DMF	10	25	37.54	0.45
PS/DMF/THF (100/0)	23	301	37.87	0.42
PS/DMF	27	715	37.90	0.39
PS/DMF	32	1080	38.51	0.34
PS/DMF	40	1210	40.21	0.30
PS/DMF/THF (75/25)	23	321	33.92	0.29
PS/DMF/THF (50/50)	23	343	32.28	0.28
PS/DMF/THF (25/75)	23	357	30.71	0.14
PS/DMF/THF (0/100)	23	362	28.97	—

^a^ Value reported for 25 °C. The ratio represented the weight ratio of DMF to THF.

**Figure 2:**
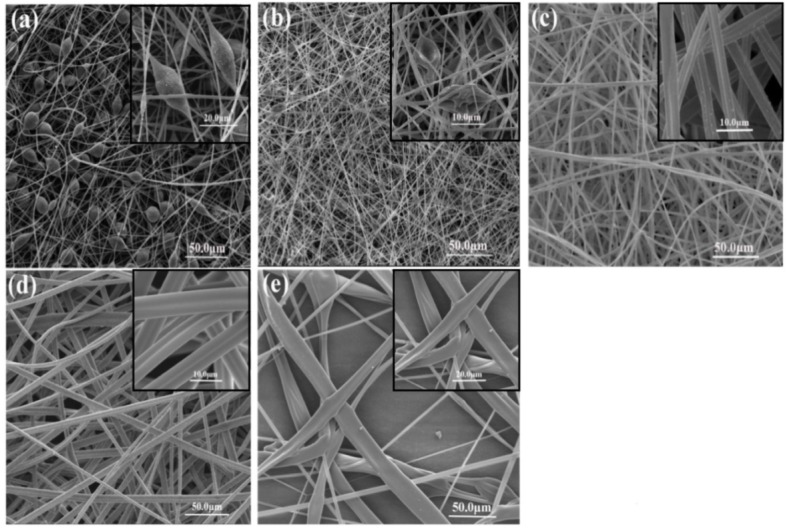
Scanning electron micrographs of electrospun PS fibers on stationary Al foil of 80 rpm, 23 (w/v)% solution concentration and applied voltage of 15 kV with different solvent combinations (**a**) DMF/THF = 100/0; (**b**) DMF/THF = 75/25; (**c**) DMF/THF = 50/50; (**d**) DMF/THF = 25/75; (**e**) DMF/THF = 0/100.

Figure 2 presents the fiber morphology variation with an increasing amount of THF in the mixture of DMF/THF. Figure 2a shows the bead-on-string structure with different fiber sizes and aspect ratios of beads formed with 23% (w/v) PS/DMF. In Figure 2b, when THF was added to the solvent at 25%, bead-on-string fibers were gradually tuned to a collapsed bead surface. The formation of beads could be related to insufficient resistance of the electrospinning solutions to resist electrical force stretching caused by low viscosity, high surface tension of the solutions and high boiling point of the solvent [24,27]. Table 1 shows that the solvent DMF has a higher boiling point of 152.8 °C than THF (66 °C), and Table 2 shows that the solution of 23% (w/v) PS/DMF/THF yielding bead-on-string morphology had the lowest viscosity of 301 mPa·s and the highest surface tension of 37.87 mN·m^−1^ compared to the other four solutions. From Figure 2c, it can be seen that the formed PS fibers are uniform without beads. The magnified SEM image in Figure 2c clearly shows that parallel double-strand fibers with a rough surface were manufactured in the solutions having an equal amount of DMF and THF. In Figure 2d, as the ratio of THF increased to 75%, smooth parallel double-strand fibers were clearly produced. It is attributed to the fact that with increasing the addition amount of THF, a lower surface tension and boiling point, as well as a higher viscosity of polymer solutions could be obtained, leading to significant resistance to sustain the elongation of polymer chains. Therefore, the collapsed bead-on-string morphology was transformed to homogeneous fibers without beads. Additionally, for the formation of parallel double-strand fibers, a possible mechanism is proposed. It is known that increasing the amount of THF in the solution can decrease the charge density due to its lower conductivity than that of DMF, leading to a trend of stable whipping and larger fiber diameter during electrospinning. It is also known that stable whipping tends to form well-organized fibers. When the solution with increased THF amount sprays out from the nozzle in a stable manner, the low surface tension and high viscosity of the solution provides less resistance to form continuous orderly fibers. Moreover, different evaporation rates of THF and DMF could result in different accumulation rates of fibers [28], namely producing a spatial fluctuation fiber structure. As a result, PS fibers with larger parallel double-strand morphology can be manufactured with proper ratios of DMF to THF, manifesting a controllable manipulation of PS fiber morphology by regulating the properties of electrospinning solutions.

In order to obtain bead-free fibers, reducing surface tension might be an appropriate approach. However, it should be applied with caution. It is not necessary that a lower surface tension of solvent will always be more suitable for electrospinning. Interestingly, it was observed in Figure 2e that flat ribbon fibers with a wide range of diameters were produced with the solvent THF due to its lower surface tension compared to the other solvents. The fiber diameter had a wide range from 3.68–19.46 μm. This result is ascribed to the higher viscoelastic force of the PS/THF solution (362 mPa·s) and low boiling point of THF (66 °C). Since jet splitting is difficult when the viscoelastic force is too large, the average fiber diameters had a wide range of values.

**Figure 3:**
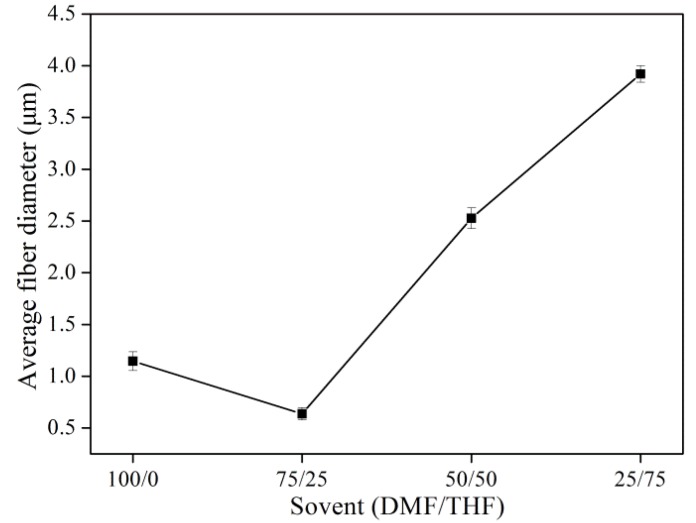
Average fiber diameter of electrospun PS fibers with different solvent combinations.

Figure 3 shows the average diameter of electrospun PS fibers prepared with different solvent combinations. The addition of 25% THF reduced the average fiber diameter from 1.14 to 0.64 μm. However, the average fiber diameter markedly increased from 0.64 to 3.92 μm with further increase of the amount of THF due to its lower surface tension of 30.71 mN·m^−1^ and higher viscosity of 357 mPa·s.

#### 3.1.2. Effect of Solution Concentration

**Figure 4:**
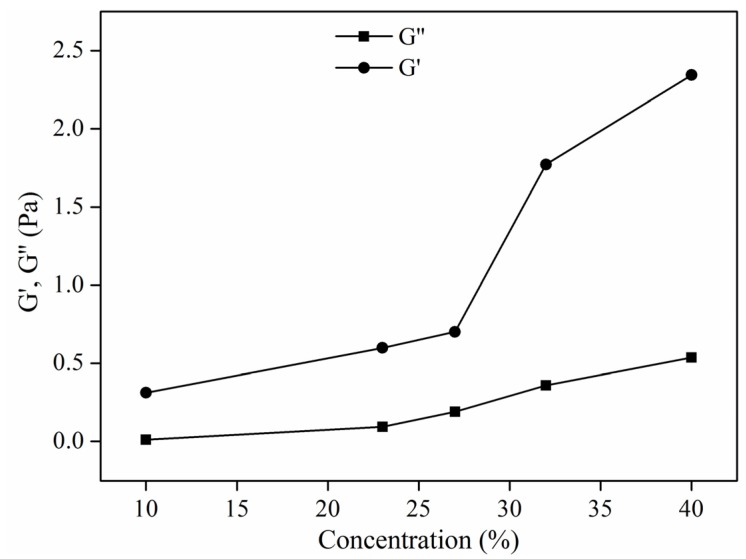
Viscoelasticity (G′, G′′) of PS solutions with different concentrations.

Solution concentration is one of the most important parameters for electrospinning and mainly affects solution viscosity. It is known that viscosity is the characterization of the intermolecular interactions in polymer solutions. The intermolecular interaction in a polymer-solvent system is either attractive or repulsive, depending on the type of solvent. Therefore, solution viscosity has a large effect on electrospinning. According to the results in Table 2, an increase in the solution concentration increased the viscosity value of the solution from 25 to 1210 mPa·s, whereas it did not significantly affect the value of the surface tension and conductivity. Figure 4 shows G′ and G′′ of the spinning solutions with different concentrations at an angler frequency of 2.5 Hz. It can be seen that G′ and G′ both increased with increasing solution concentration but the difference between G′ and G′ was larger at higher concentration than that at lower concentration, indicating that the elastic property of PS solution was more significant at high concentrations. This is attributed to the fact that at higher concentration, the number of PS chains per unit volume of solvent is much more than that at lower concentration, resulting in enhanced interaction and entanglement of PS chains to resist deformation.

**Figure 5:**
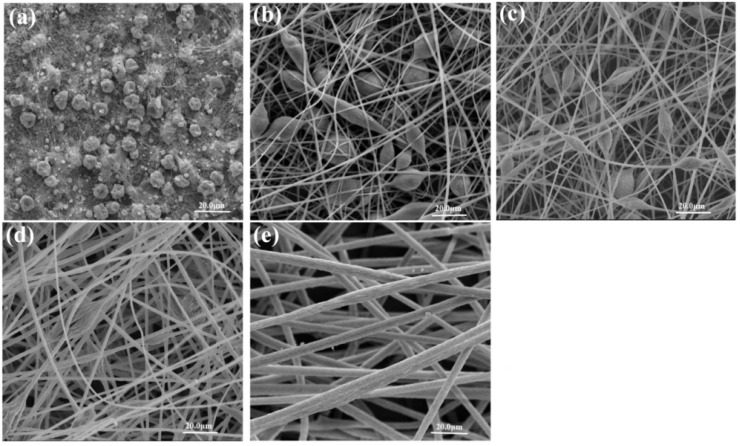
Scanning electron micrographs of electrospun PS fibers on stationary Al foil of 80 rpm and applied voltage of 15 kV with different concentrations of PS/DMF solutions (**a**) 10% (**b**) 23% (**c**) 27% (**d**) 32% (**e**) 40% (w/v).

Figure 5 exhibits SEM photographs of electrospun PS fiber prepared from PS/DMF solutions at concentrations of 10%, 23%, 27%, 32%, and 40% (w/v). The amount of beads decreased with increased polymer concentration, and eventually formed straight fibers. It was observed in Figure 5a for the diluted solution of 10% (w/v) that irregular beads were formed because the very low viscosity did not suffice to sustain the elongation of the liquid jet, and therefore the thin jet of solution left the nozzle instantly and shrunk to droplets. Moreover, at 10% (w/v), PS solution exhibited typical viscoelastic property, which means that elasticity is too small to provide resistance to sustain the elongation resulting from electrostatic force, leading to bead morphology. With further increase to concentrated solutions of 23%–32% (w/v), ultrafine fibers were formed through beads as shown in Figure 5b–d. This is because the high viscosity and polymer content cause the solution jet to elongate and solidify quickly. More importantly, the difference between G′ and G′′ was larger at 23%–32%, leading to the fact that the elasticity gradually turned to be a key role in the PS solution’s rheological behavior (shown in Figure 4), and the larger elasticity could offer more resistance to sustain elongation during the initial electrospinning stage. However, the inherent viscous characteristic could also contribute to the unrecoverable deformation of PS at these concentrations, partially counteracting the elastic effect. Thus, the enhanced resistance against electrostatic force can produce fibers with bead-on-string structure. As the concentration increases to 40%, the combination of viscosity and viscoelastic property of the PS solution was large enough to favor fiber formation, shown in Figure 5e.

**Figure 6:**
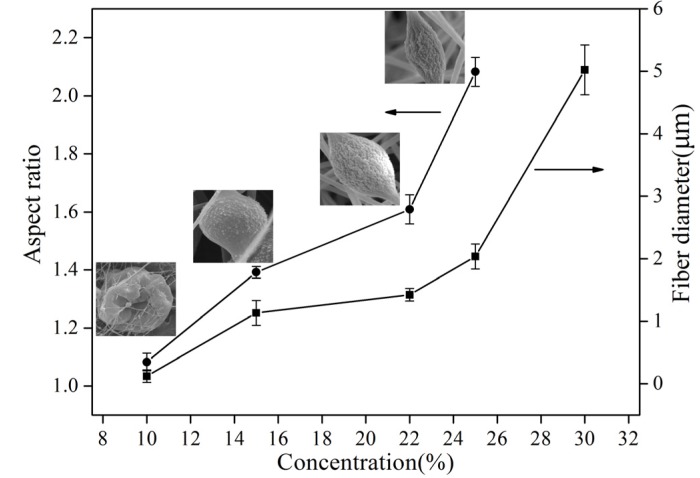
Aspect ratio of beads and fiber diameter of PS fibers with different concentrations.

Figure 6 shows the aspect ratio of beads and fiber diameters as a function of the polymer concentration. The aspect ratio and fiber diameter increased proportionally with the increase of polymer concentration. In order to explain the effect of solution concentration on the diameters or widths of the as-spun fibers, analysis of all forces acting on a small segment of a charged jet is necessary. Jarusuwannapoom *et al.* [18] summarized six types of forces to be considered: gravitational force, electrostatic force, Coulombic force, viscoelastic force, surface tension, and drag force. Among these forces, only the Coulombic, the viscoelastic, and the surface tension forces are responsible for the formation of beads as well as the shrinking of the charged jet during its flight to the grounded target [18]. As to the polymer solution, solvent molecules tend to aggregate and form a spherical shape due to the effect of surface tension at lower concentration which has more solvent fraction. The viscosity increase with the concentration of polymer illustrates the stronger effect on the interaction between polymeric chains and solvent. Consequently, solvent molecules tend to make the entangled molecular chains separate to reduce the tendency for aggregation and shrinkage.

#### 3.1.3. Effect of Spinning Voltage

**Figure 7:**
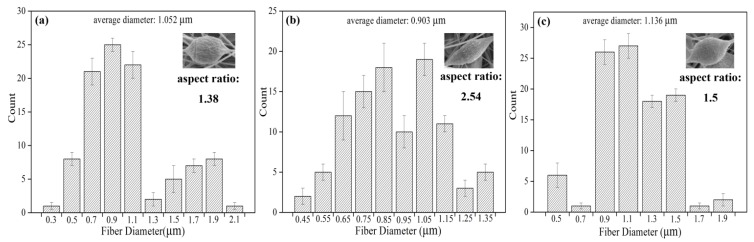
Fiber diameter distributions of electrospun 20 wt% PS fibers with different applied voltages (**a**) 10 kV (**b**) 15 kV (**c**) 20 kV.

As the applied voltage directly affects the dynamics of the liquid flow, the changes in the voltage reflect on the shape of the suspending droplets out of the nozzle of the spinneret, on its surface charge, dripping rate, and especially the structural morphology (*i.e.*, fiber diameter) of the electrospun fibers. The jet diameter becomes smaller as it travels to the ground, attributed to two factors: solvent evaporation and continuous stretching caused by electrical force. Although some researchers have shown that the applied voltage has less effect on the bead formation and fiber diameter than the other parameters studied above [29], certain conclusions can still be reached in this study. Figure 7 shows the resulting bead morphology, aspect ratio and average fiber diameter of PS/DMF electrospun fibers at varying voltage ranges. Three applied voltages of 10, 15 and 20 kV were individually applied for electrospinning 23% (w/v) PS/DMF solutions while keeping all other parameters constant, setting the collection distance to15 cm, and the rotational speed at 80 rpm. Figure 7a–c show that as the applied voltage increased, the average fiber diameter increased. The applied voltage of 20 kV in Figure 7c made the polymer solution eject in a more fluid jet and resulted in an average fiber diameter of 1.136 μm, while the voltages of 15 kV and 10 kV yielded average diameters of 0.903 μm and 1.052 μm, respectively. From these results, it is interesting to note that the distribution of the fiber diameters was very broad when the applied voltage was 10 kV, was quite narrow at the applied potentials of 15 kV, and was broad again at the applied potential of 20 kV. Another interesting point is the effect of applied potential on the morphology of the obtained beads. Figure 7 clearly shows that the aspect ratio of beads formed at 15 kV was much higher than those of the beads formed at 10 and 20 kV.

The effect of applied voltage on the fiber diameters can be explained in terms of the relationships between the three major forces (the Coulombic, the viscoelastic and the surface tension), which influence the fiber diameters. At low applied potentials (e.g., 10 kV), the Coulombic force was not high when compared with that of the surface tension. This resulted in as-spun fibers with large diameters and the presence of large beads along the fibers. At a moderate applied potential (e.g., 15 kV), all three forces were well-balanced, resulting in a narrow distribution of the fiber diameters. With further increase in the applied potential (e.g., 20 kV), the Coulombic force was much greater than the viscoelastic force. This might result in a higher possibility of breakage of an over-stretched charged jet during its flight to the target. Moreover, with an increased applied potential, a charged jet travelled to the grounded target much faster. The solvent, therefore, had less time to evaporate. The charged jet retracts and some of the jets were neutralized, which led to bigger but irregular fibers.

### 3.2. Fiber Mat Wettability

The water contact angle values of the PS films, which were fabricated by solvent evaporation on an aluminum plate from the solution of PS in DMF and THF with a concentration of 23% (w/v), were 106 ± 1.5° and 90 ± 1.2°, respectively. It is well known that PS is chemically hydrophobic. Therefore its water contact angle is higher than those of other hydrophilic polymers. However, the value of the water contact angle is not sufficient enough to meet the superhydrophobic requirements for many practical applications. In addition, the surface morphology of electrospun mats can be designed and controlled during the electrospinning process. Therefore, it is reasonable to deduce that taking advantage of the hydrophobic behavior of PS and the special characteristic of the electrospinning technique, electrospun mats with various PS fibers morphologies can be prepared with good hydrophobicity.

**Figure 8:**
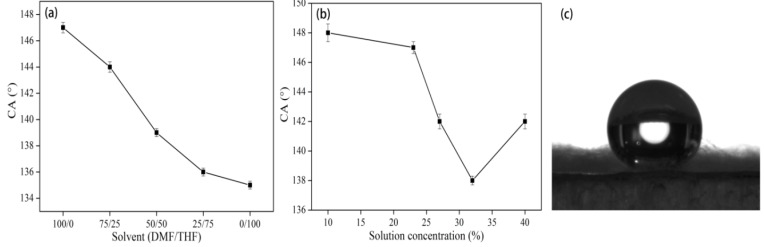
Contact angle of electrospun PS nanofibrous mats with (**a**) different solvent combinations and (**b**) different concentrations (**c**) water droplet on electrospun PS fibers from 23% (w/v) PS/DMF, CA = 147°.

The water contact angles of various electrospun PS mats fabricated with different solvents and concentrations are shown in Figure 8. The CA value decreased with increasing amount of THF in the solvent. Moreover, Figure 8b shows that the CA value could be significantly affected by solution concentration and the smallest CA value was found using concentration of 32% (w/v). From Figure 8a,b, it can also be seen that the CA value of 23% (w/v) PS/DMF is as high as 147°, which is much higher than those of the films resulting from solvent evaporation, indicating that the electrospun PS mats are sufficient enough to exhibit superhydrophobicity in practical applications. The actual behavior of a water droplet on the surface of electrospun PS fiber is shown in Figure 8c. To our knowledge, the surface structure of the electrospun mat plays a leading role in determining the hydrophobicity. Moreover, the electrospun fibers with bead, porous and protuberant structure also contribute to the roughness of the electrospun mat. More air can be trapped as the roughness of the PS surface increases, which is beneficial to improve the hydrophobicity of the membranes as the water CA of air is considered to be 180° based on the Cassie equation [30]. Therefore, it is believed that beads have a predominant effect on the resultant hydrophobic behavior of the electrospun mat, and the fibers with wide diameter range are with the limitation of advancing superhydrophobicity. Based upon the illustrated effect of solvents above, with more THF added, the amount of beads decreased gradually and the rough surface of the electrospun products turned smooth. Furthermore, the effect of solution concentration demonstrates that beads morphology can be easily produced when the solution concentration is low. Thus, the CA value gradually decreases with the bead-on-string fiber turning to fibers, which is shown in Figure 8a,b.

**Figure 9:**
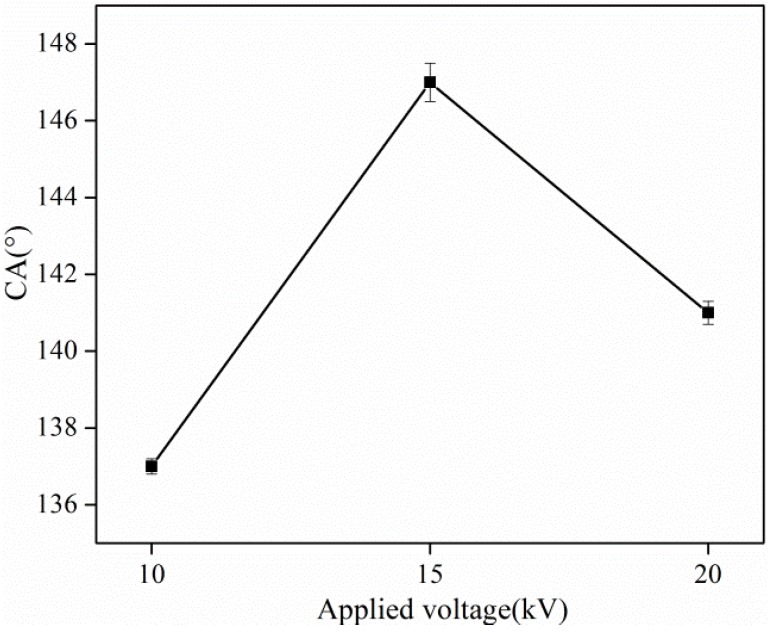
Contact angle of electrospun PS nanofibrous mats with different applied voltages.

Figure 9 presents the contact angles of PS mats made out of different fibers prepared using different applied voltages. In Figure 9, it is shown that the CA value of mats with their fibers prepared using an applied voltage of 15 kV is up to 147°, while the other two values using 10 kV and 20 kV are 137° and 141°, respectively. During the electrospinning process, the diameter of fibers prepared using an applied voltage of 15 kV is much larger than the other two according to Section 3.1.3, while the CA of 15 kV is also the largest as shown in Figure 9. On other hand, large diameter fibers are not favorable for increasing the roughness to form a better hydrophobic surface. Since the electrospun nanofibers had a small diameter, consequently the water droplet came into contact with a relevant small area of the electrospun mat compared with other materials and resulted in smaller CA values.

### 3.3. Fiber Mat Dynamic Mechanical Property

**Figure 10:**
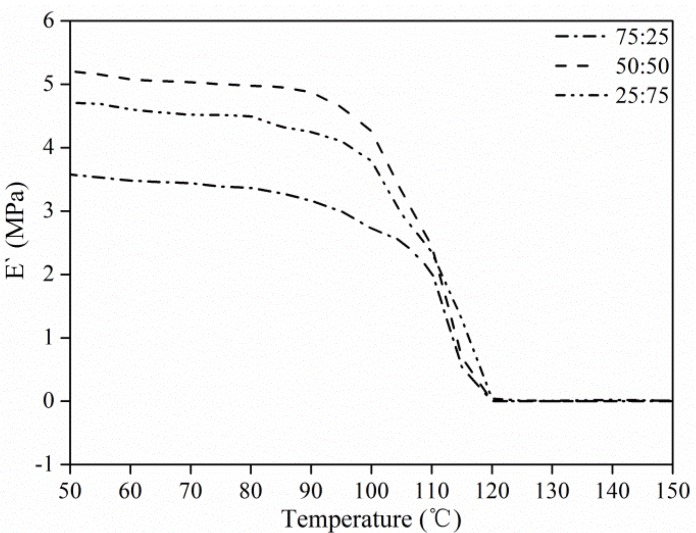
Dynamic mechanical analyzer (DMA) curves of electrospun PS nanofibrous mats.

The storage modulus (E′) of the electrospun double-strand PS fibrous mats is shown in Figure 10. All the curves represent a typical change in E′ as temperature increases, of an amorphous, high-molecular weight thermoplastic polymer. For temperatures below the glass transition region, the E′ of these mats decreased slightly with temperature because the PS was in the glassy state and the molecular motions were largely restricted to vibration and short-range rotation. The corresponding viscoelastic relaxation phenomenon at the transition region was observed at 100 °C and produced a drastic drop in E′ due to unrecoverable melting deformations of the polymer matrix. DMA curves clearly showed that the double-strand structure could significantly reinforce the E′ of PS fibrous mats, producing the maximum E′ of approximately 5.13 MPa. This reinforcing effect can be attributed to the further restriction in mobility of PS fibers by the rough double-strand structure with chain friction and entanglement.

### 3.4. Fiber Mat Tensile Strength

**Figure 11:**
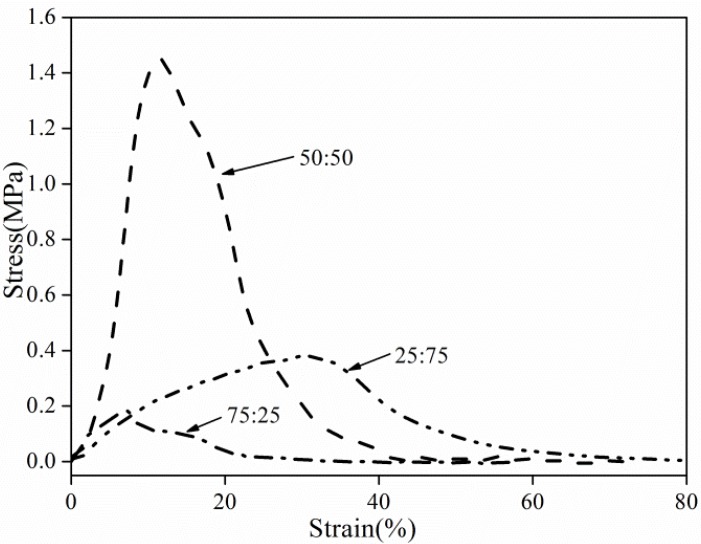
Stress-strain curves of electrospun PS nanofibrous mats.

The tensile properties of electrospun mats with a double-strand structure are shown in Figure 11. The mechanical property of electrospun mats was enhanced with the presence of double-strand fibers. As bead-on-string fibers appeared using solvent of DMF/THF at a ratio of 75/25 in the prepared mat, the mat demonstrates a weaker tensile strength of less than 0.2 MPa. This behavior is mainly attributed to the unique characteristic of PS. Electrospun PS mats are generally soft and flexible. When the weight ratio of THF increased, double-strand fibers with rough surface formed. The tensile strength of the formed mats was almost 1.5 MPa, which indicated that the presence of double-strand fibers contributed to the reinforcement of electrospun PS mats and overcame the inherent shortcomings of PS mats. This result could be explained by the fact that during the tensile test, the friction effect and close compacting of double-strand fibers with rough surface provided effective neutralization to the stress and partially dissipated the tensile energy, resulting in an increase in stress and a corresponding decrease in strain. However, the tensile strength decreased to 0.4 MPa as THF increased, attributed to the reduced dissipation effect of the smooth fiber surface.

## 4. Conclusions

In the present work, the effects of solvents, solution concentrations and applied voltage on the electro-spinnability of PS solutions along with the morphological appearance of the as-spun PS fibers and nanofibrous mat properties, were all characterized. Various surface morphologies, including beads with different sizes and shapes, bead-on-string structure with different aspect ratios of the beads, as well as fibers with different diameters and shapes, were formed by tuning the physical properties of the solvents and PS solutions. High surface tension, low viscosity, and typical viscoelasticity of the polymer solution contribute to the formation of the bead structure. A new kind of fiber with a double-strand structure was formed at moderate viscosity by using a mixed solvent of DMF and THF. It was also found that the increased viscosity and the typical elasticity characteristic of the polymer solution led to a tendency to form fibers. Distribution of fiber diameter was quite broad when the applied voltage was 10 and 20 kV, but narrowed with a voltage of 15 kV. The maximum water CA value of electrospun PS films obtained from DMF at 15 kV reached 148°. The DMA results showed that the double-strand could significantly reinforce the storage modulus of PS nanofibrous mats. The tensile strength of electrospun double-strand fibers with rough surfaces was 1.5 MPa, while the corresponding tensile strength of the mats with smooth surfaces was only 0.4 MPa.

## References

[1] Lu X., Wang C., Wei Y. (2009). One-dimensional composite nanomaterials: Synthesis by electrospinning and their applications. Small.

[2] Sun B., Long Y.Z., Zhang H.D., Li M.M., Duvail J.L., Jiang X.Y., Yin H.L. (2014). Advances in three-dimensional nanofibrous macrostructures via electrospinning. Prog. Polym. Sci..

[3] Reneker D.H., Yarin A.L., Fong H., Koombhongse S. (2008). Bending instability of electrically charged liquid jets of polymer solutions in electrospinning. Polymer.

[4] Greiner A., Wendorff J.H. (2007). Electrospinning: A fascinating method for the preparation of ultrathin fibers. Angew. Chem. Int. Ed. Engl..

[5] He D., Hu B., Yao Q.F., Wang K., Yu S.H. (2009). Large-scale synthesis of flexible free-standing SERS substrates with high sensitivity: Electrospun PVA nanofibers embedded with controlled alignment of silver nanoparticles. ACS Nano.

[6] Park S.M., Kim D.S. (2015). Electrolyte-assisted electrospinning for a self-assembled, free-standing nanofiber membrane on a curved surface. Adv. Mater..

[7] Santos C., Silva C.J., Büttel Z., Rodrigo G., Sara B.P., Paula T., Andrea Z. (2014). Preparation and characterization of polysaccharides/PVA blend nanofibrous membranes by electrospinning method. Carbohyd. Polym..

[8] Abdelgawad A.M., Hudson S.M., Rojas O.J. (2014). Antimicrobial wound dressing nanofiber mats from multicomponent (chitosan/silver-NPs/polyvinyl alcohol) systems. Carbohyd. Polym..

[9] Schreuder-Gibson H.L., Gibson P.K., Senecal M., Sennett J., Walker W., Yeomans D.Z., Tsai P.P. (2002). Protective textile materials based on electrospun nanofilbers. J. Adv. Mater..

[10] Zhu M.F., Zuo W.W., Yu H., Yang W., Chen Y.M. (2006). Superhydrophobic surface directly created by electrospinning based on hydrophilic material. J. Mater. Sci..

[11] Menini R., Farzaneh M. (2008). Production of superhydrophobic polymer fibers with embedded particles using the electrospinning technique. Polym. Int..

[12] Celia E., Darmanin T., de Givenchy E.T., Amigoni S., Guittard F. (2013). Recent advances in designing superhydrophobic surfaces. J. Colloid. Interface Sci..

[13] Krishnan K.A., Anjana R., George K.E. (2014). Effect of alkali-resistant glass fiber on polypropylene/polystyrene blends: Modeling and characterization. Polym. Compos..

[14] Ma M.L., Hill R.M., Lowery J.L., Fridrikh S.V., Rutledge G.C. (2005). Electrospun poly(Styrene-block-dimethylsiloxane) block copolymer fibers exhibiting superhydrophobicity. Langmuir.

[15] Hardman S.J., Sarih N.M., Riggs H.J., Thompson R.L., Rigby J., Bergius W.N.A., Hutchings L.R. (2011). Electrospinning superhydrophobic fibers using surface segregating end-functionalized polymer additives. Macromolecules.

[16] Bai L., Gu J.Y., Huan S.Q., Li Z.G. (2014). Aqueous poly (vinyl acetate)-based core/shell emulsion: Synthesis, morphology, properties and application. RSC Adv..

[17] Yoon Y.L., Moon H.S., Lyoo W.S., Lee T.S., Park W.H. (2009). Superhydrophobicity of cellulose triacetate fibrous mats produced by electrospinning and plasma treatment. Carbohyd. Polym..

[18] Jarusuwannapoom T., Hongrojjanawiwat W., Jitjaicham S., Wannatong L., Nithitanakul M., Pattamaprom C., Koombhongse P., Rangkupan R., Supaphol P. (2005). Effect of solvents on electro-spinnability of polystyrene solutions and morphological appearance of resulting electrospun polystyrene fibers. Eur. Polym. J..

[19] Kang M., Jung R., Kim H.S., Jin H.J. (2008). Preparation of superhydrophobic polystyrene membranes by electrospinning. Colloid. Sur. A.

[20] Zhan N., Li Y., Zhang C., Song Y., Wang H., Sun L., Yang Q., Hong X. (2010). A novel multinozzle electrospinning process for preparing superhydrophobic PS films with controllable bead-on-string/microfiber morphology. J. Colloid. Interface Sci..

[21] Uyar T., Besenacher F. (2008). Electrospinning of uniform polystyrene fibers: The effect of solvent conductivity. Polymer.

[22] Zheng J.F., He A.H., Li J.X., Xu J., Han C.C. (2006). Studies on the controlled morphology and wettability of polystyrene surfaces by electrospinning or electrospraying. Polymer.

[23] Fong H., Chun I., Reneker D.H. (1999). Beaded nanofibers formed during electrospinning. Polymer.

[24] Doshi J., Reneker D.H. (1995). Electrospinning process and applications of electrospun fibers. J. Electrostat..

[25] Rojas O.J., Montero G.A., Habibi Y. (2009). Electrospun nanocomposites from polystyrene loaded with cellulose nanowhiskers. J. Appl. Polym. Sci..

[26] Marie R.L., Christian P. (2015). Partial disentanglement in continuous polystyrene electrospun fibers. Macromolecules.

[27] Fong H., Reneker D.H. (1999). Elastomeric nanofibers of styrene–butadiene–styrene triblock copolymer. J. Polym. Sci. B Polym. Phys..

[28] Wang L.F., Pai C.L., Boyce M.C., Rutledge G.C. (2009). Wrinkled surface topographies of electrospun polymer fibers. Appl. Phys. Lett..

[29] Jacobs V., Anandjiwala R.D., Maaza M. (2010). The influence of electrospinning parameters on the structural morphology and diameter of electrospun nanofibers. J. Appl. Polym. Sci..

[30] Cassie A.B.D., Baxter S. (1944). Wettability of porous surfaces. Trans. Faraday Soc..

